# Novel insights into endothelial cell malignancies

**DOI:** 10.18632/oncotarget.26516

**Published:** 2018-12-25

**Authors:** Taekyu Ha, Hidetaka Ohnuki, Giovanna Tosato

**Affiliations:** Giovanna Tosato: Laboratory of Cellular Oncology, Center for Cancer Research, National Cancer Institute, National Institutes of Health, Bethesda, MD, USA

**Keywords:** endothelial cells, endothelial cell tumors, angiosarcoma, DLC1, YAP/TAZ

Endothelial cells contribute to tumor growth through sprouting angiogenesis and formation of new blood vessels in response to inductive signals from the tumor cells and the tumor microenvironment. Tumor-derived pro-angiogenic signals and endothelial cell responses to these signals are similar to those operational in physiological angiogenesis, but their magnitude and duration are abnormally increased. This is linked to exaggerated vessel sprouting and abnormal morphology and function of tumor vessels but does not lead to endothelial cell transformation.

Malignancies of endothelial cell derivation are rare; they include aggressive angiosarcomas that are locally invasive and metastasize early, and the less aggressive Kaposi’s sarcoma (KS) and epithelioid hemangioendotheliomas. KS is a viral malignancy caused by Kaposi’s sarcoma-associated herpesvirus (KSHV; also called human herpesvirus-8, HHV8) [1]. There are different epidemiologic forms of KS: “classic” KS affecting elderly man in the Mediterranean regions; “endemic” KS affecting people in sub-Saharan Africa; “transplant-associated” KS; and AIDS-associated or “epidemic” KS. This tumor is characterized by multiple vascular-type lesions involving the lower legs, the oral mucosa, the gastrointestinal tract, the lungs and other organs. Typically, the lesions are composed of KSHV-infected elongated (spindle)-type cells of endothelial derivation, which form abnormal, blood-rich vascular structures, mixed with a variable number of inflammatory cells and fibrosis. The virus is mostly latent in KS but undergoes activation of lytic genes and sometimes viral replication. The viral latency-associated nuclear antigen (LANA) and the lytic G-coupled protein receptor (vGPCR) are critical contributors to KS development and progression, in part because they cause nuclear accumulation of hypoxia-inducible factor 1a (HIF-1α), a transcriptional regulator of pro-survival and pro-angiogenic genes [2]. vGPCR also activates the transcriptional co-activators YAP/TAZ by inhibiting the Hippo pathway kinases LATS1/2, which normally inactivate YAP/TAZ [3]. The successful treatment of KS in immunodeficient patients [1] generally requires control of the underlying immunodeficiency. In patients with AIDS, control of HIV infection by itself often leads to KS responses. In non-responsive patients, particularly those with extensive disease, systemic therapy with pegylated liposomal doxorubicin, paclitaxel and other drugs have been successful.

Unlike KS, the etiology of epithelioid hemangioendotheliomas and angiosarcomas is unclear. Epithelioid hemangioendotheliomas are extremely rare and difficult to diagnose [4, 5]; the lesion or lesions (in some cases hemangioendotheliomas present as multifocal lesions without a preferential anatomic location) are composed of CD31^+^ endothelial cells assembled in poorly-defined vascular channels separated by abundant extracellular matrix, with various degrees of mitotic activity and infiltration of surrounding tissue. Epithelioid hemangioendothelioma has a reported 5-year overall survival of 73% [4]. Interestingly, genetic studies have identified gene fusions of *TAZ* or *YAP* genes in epithelioid hemangioendotheliomas, including a *TAZ-CAMTA1*, *TAZ-FOSB* or *YAP1-TFE3* gene fusions [4]. All proteins derived from these *YAP/TAZ* gene fusions retain the TEAD-binding domain, suggesting that TEAD-dependent transcription of oncogenic genes is active in hemangioendothelioma and contributing to malignancy [6].

Angiosarcomas are aggressive endothelial malignancies that can occur anywhere in the body (primary angiosarcoma) or arise from lymphedema at the lower extremities or in the chest wall exposed to radiation for breast cancer (secondary angiosarcoma) [5, 7]. All clinical subtypes are characterized histologically by mitotic tumor cells that express CD31 to varying degrees and form vascular structures replete of blood extravasating into the parenchyma. The tumor cells are locally invasive, often recognized at a distance from the primary tumor after resection with large margins, and highly metastatic at an early stage. Currently, the 5-year survival of patients with angiosarcoma is 33.5% [7]. Multiple genetic abnormalities have been described in angiosarcoma but no unifying driver mutation [5, 7]. Subsets of angiosarcomas show mutations of *PTPRB* coding for the protein tyrosine phosphatase receptor type B (PTBRB); of *KDR* or *FLT4* coding, respectively, for VEGFR2 and VEGFR3; and single-nucleotide changes in *PLCG1*, coding for phospholipase C, gamma 1. Diverse mutations of *KRAS*, *HRAS*, *NRAS*, *BRAF*, *MAPK1* and *NF1* have also been reported is subsets of angiosarcomas. Myc amplification has been frequently noted in radiation-induced angiosarcoma [5]. An analysis of mutation burden in 157 angiosarcoma specimens reported that the median mutation burden was 3.8 mutations/Mb, and that 13.4% of cases had more than 20 mutations/Mb is 12.3 [8]. This relatively-high mutation burden suggests that some angiosarcomas may respond to checkpoint inhibitors/other immune therapies. A remarkable tumor response was observed in one patient with angiosarcoma on anti-PD1 treatment, prompting ongoing clinical trials [7].

Recently, we have found that the tumor suppressor gene *Deleted in Liver Cancer 1* (*DLC1*) is expressed at abnormally low levels in six primary angiosarcomas compared to the normal adjacent endothelium [9]. DLC1 is a *rho*-GTPase-activating protein that inactivates *rho*-A, -B and -C; this function is critical to DLC1 tumor suppression [10]. Additionally, DLC1 establishes functional interactions with focal adhesion family members ensuring its localization to focal adhesions, which contributes to its tumor suppressive functions. Our studies aimed at investigating the potential role of DLC1 loss in angiosarcoma, took the approach of silencing DLC1 in various types of primary endothelial cells, which normally express DLC1 [9]. We found that DLC1-depleted endothelial cells lose “cell contact inhibition of growth”, i.e. the property of normal cells to stop growing and begin to die when confluent, which is fundamental to regulation of precise tissue and organ size. Loss of cell contact inhibition is a hallmark of tumor cells. Our initial experiments could not fully identify the mechanisms underlying DLC1 regulation of cell-contact growth inhibition.

**Figure 1:**
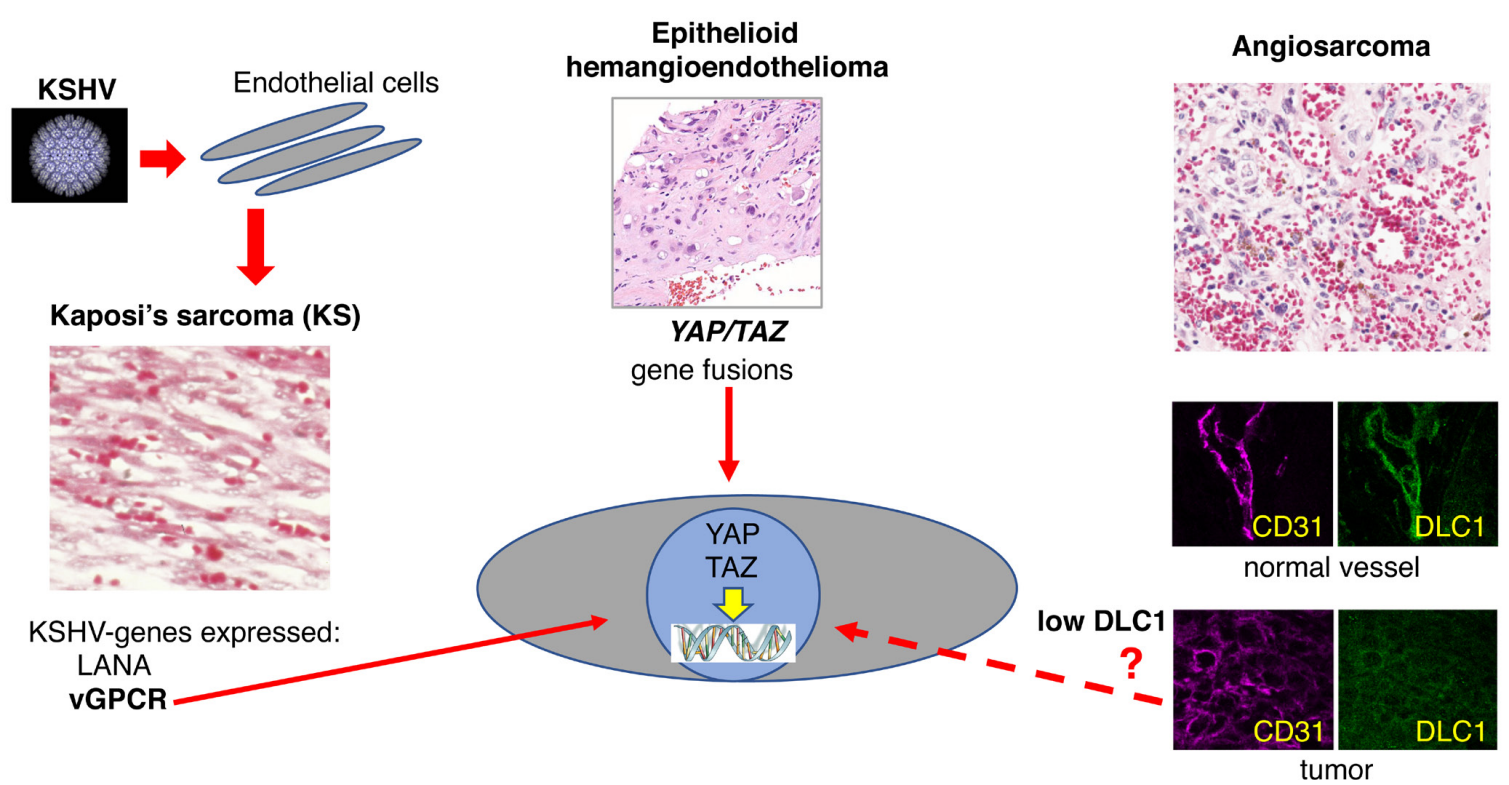
Pathways of endothelial transformation KSHV-infected endothelial lineage cells in KS express the oncogenic viral genes LANA and vGPCR, which activates nuclear YAP and TAZ. Activating fusions of *YAP* or *TAZ* are present in some epithelioid hemangioendotheliomas (image of JM Gardner). The tumor suppressor DLC1 protein is abnormally low in angiosarcomas compared to the normal endothelium; angiosarcomas contain active YAP/TAZ.

Studies by other laboratories have identified the Hippo signaling pathway and its effectors co-transcriptional activators YAP and TAZ as critical regulators of cell contact inhibition of growth and organ size [6]. Many cancer cells have hyperactive YAP and TAZ, including KS and angiosarcoma cells. Based on this knowledge, we are currently examining if DLC1 deficiency is an upstream inducer of YAP/TAZ in angiosarcoma, and if hyperactive YAP/TAZ may represent a unifying link in endothelial cell oncogenesis (Figure 1). In KS, the YAP/TAZ inducer has been identified as KSHV-vGPCR [3]. Some epithelioid hemangioendotheliomas display activating mutations of YAP and TAZ genes [4]. If it turns out that hyperactive YAP/TAZ are downstream mediators of DLC1 deficiency, it would open an exciting treatment opportunity for angiosarcoma because YAP/TAZ targeting is currently under development.
